# Does the Serum Concentration of Angiotensin II Type 1 Receptor Have an Effect on the Severity of COVID-19? A Prospective Preliminary Observational Study among Healthcare Professionals

**DOI:** 10.3390/jcm11071769

**Published:** 2022-03-23

**Authors:** Jarosław Janc, Michał Suchański, Magdalena Mierzchała-Pasierb, Ewa Woźnica-Niesobska, Lidia Łysenko, Patrycja Leśnik

**Affiliations:** 1 Department of Anaesthesiology and Intensive Therapy, 4th Military Clinical Hospital, 50-981 Wroclaw, Poland; michal.suchanski@icloud.com (M.S.); patrycja.lesnik@gmail.com (P.L.); 2 Department of Medical Biochemistry, Wroclaw Medical University, 50-369 Wroclaw, Poland; magdalena.mierzchala-pasierb@umw.edu.pl; 3 Department of Anaesthesiology and Intensive Therapy, Wroclaw Medical University, 50-556 Wroclaw, Poland; ewa.anna.woznica@gmail.com (E.W.-N.); lidia.lysenko@umw.edu.pl (L.Ł.)

**Keywords:** SARS-CoV-2, COVID-19, angiotensin 1 receptor (AT1R), AT1R concentration, angiotensin II, symptoms’ severity

## Abstract

SARS-CoV-2 is a virus that causes severe respiratory distress syndrome. The pathophysiology of COVID-19 is related to the renin–angiotensin system (RAS). SARS-CoV-2, a vector of COVID-19, uses angiotensin-converting enzyme 2 (ACE-2), which is highly expressed in human lung tissue, nasal cavity, and oral mucosa, to gain access into human cells. After entering the cell, SARS-CoV-2 inhibits ACE-2, thus favouring the ACE/Ang II/angiotensin II type 1 receptor (AT1R) axis, which plays a role in the development of acute lung injury (ALI). This study aimed to analyse the influence of angiotensin 1 receptor (AT1R) levels in the serum on the course of the severity of symptoms in healthcare professionals who had a SARS-CoV-2 infection. This prospective observational study was conducted on a group of 82 participants. The study group included physicians and nurses who had a COVID-19 infection confirmed by real-time reverse transcription-polymerase chain reaction (RT-PCR) test for SARS-CoV-2. The control group consisted of healthy medical professionals who had not had a SARS-CoV-2 infection or who had no symptoms of COVID-19 and who tested negative for SARS-CoV-2 on the day of examination. We analysed the correlation between AT1R concentration and the severity of COVID-19, as well as with sex, age, blood group, and comorbidities. There were no statistically significant differences in the mean values of AT1R concentration in the recovered individuals and the non-COVID-19 subjects (3.29 vs. 3.76 ng/mL; *p* = 0.32). The ROC curve for the AT1R assay showed an optimal cut-off point of 1.33 (AUC = 0.44; 95% CI = 0.32–0.57; *p* = 0.37). There was also no correlation between AT1R concentration and the severity of symptoms associated with COVID-19. Blood type analysis showed statistically significantly lower levels of AT1R in COVID-19-recovered participants with blood group A than in those with blood group O. In conclusion, AT1R concentration does not affect the severity of symptoms associated with COVID-19 among healthcare professionals.

## 1. Introduction

Coronavirus Disease 2019 (COVID-19) was declared a world pandemic by the World Health Organization (WHO) on 11 March 2020 [1]. Since then, the understanding of COVID-19 pathophysiology and therapeutic options has evolved. The incubation period of COVID-19 is estimated to be 14 days from exposure, with a median time of 4–5 days [2], and the severity of the disease may range from asymptomatic to severe pneumonia with acute respiratory distress syndrome (ARDS). The symptoms of coronavirus type 2 (SARS-CoV-2) infection and the severity of COVID-19 are evaluated according to the illness categories described in the Clinical Spectrum of SARS-CoV-2 Infection section of the COVID-19 Treatment Guidelines developed by the National Institutes of Health (NIH) [3].

An asymptomatic or presymptomatic infection is described in individuals with a positive virologic test for SARS-CoV-2 but without symptoms consistent with COVID-19. Symptoms such as fever, cough, sore throat, muscle pain, diarrhoea, loss of smell and taste, malaise, and fatigue are classified as mild illness [3]. Patients with lower respiratory disease with a saturation of oxygen (SpO_2_) ≥ 94% in room air at sea level are classified as having a moderate illness, and those with SpO_2_ < 94% and PaO_2_/FiO_2_ < 300 mmHg as having a severe illness. A critical illness is described in individuals with respiratory failure, septic shock, and/or multiple organ dysfunction [3].

The pathophysiology of COVID-19 is related to the renin–angiotensin system (RAS). SARS-CoV-2, a vector of COVID-19, uses angiotensin-converting enzyme 2 (ACE-2), which is highly expressed in human lung tissue, nasal cavity, and oral mucosa, to gain access into human cells [4].

**Figure 1:**
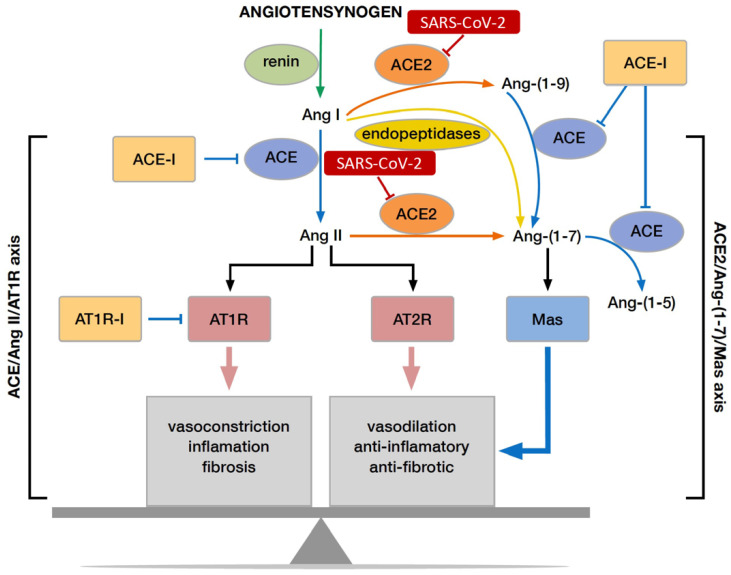
Influence of SARS-CoV-2 on RAS pathway.

The substrate of the RAS pathway, angiotensinogen, is released into the circulation by the liver, where it is cleaved by an enzyme, renin, into angiotensin I (AngI) [5]. AngI is converted into angiotensin II (AngII) by ACE upon entering the ACE/AngII/AT1R axis of RAS. AngI and AngII are converted by ACE-2 into Ang1–9 and Ang1–7, respectively, which are the molecules of the ACE-2/AT2R axis. AngII, by interacting with its receptor– AngII receptor type 1 (AT1R), causes vasoconstriction, cell proliferation, hypertrophy, fibrosis, and inflammation [6]. By contrast, its interaction with AT2R is responsible for counterbalancing the effects of AT1R activation [7]. After entering the cell, SARS-CoV-2 inhibits ACE-2, thus favouring the ACE/AngII/AT1R axis, which plays a role in the development of acute lung injury (ALI) [8]. Elevated AngII levels have been determined in ALI and correlated with the severity and mortality of the disease (Figure 1) [9,10].

AngII/AT1R activation leads to endothelial dysfunction and the activation of the coagulation cascade. AngII/AT1R acts by increasing reactive oxidative species (ROS) and promoting inflammation, inter alia leading to an increase in C-reactive protein (CRP) and interleukin-6 (IL-6) levels; such changes are observed in COVID-19 infection and are considered predictors of disease severity [11]. The formation of ROS results in the production of inflammatory factors, such as tumour necrosis factor-alpha (TNF-α), monocyte chemoattractant protein-1 (MCP-1), tissue factor (TF), nuclear factor kappa B (NF-κB), IL-6, CRP, and plasminogen activator inhibitor-1 (PAI-1), which may add to the state of overwhelming systemic inflammation, and hypercoagulability [12]. Further, increased aldosterone release, mediated by AngII/AT1R, may be associated with thrombotic events [13]. It should be emphasised that AngII and aldosterone increase the expression of PAI-1, a major inhibitor of fibrinolysis in vivo, in vascular smooth muscle and endothelial cells [14]. Aldosterone release, stimulated by AngII/AT1R activation, also upregulates protein-C receptors in the human vascular endothelium [15] and is strongly associated with a prothrombotic state [16].

This study aimed to determine the correlation between AT1R serum concentration and the severity of SARS-CoV-2 in healthcare professionals who work with patients with COVID-19.

## 2. Materials and Methods

### 2.1. Design and Settings

The single-centre prospective observational study was conducted in January 2021 at the Department of Anaesthesiology and Intensive Therapy of the 4th Military Clinical Hospital in Wroclaw, Poland. The study was prospectively registered in the Australian New Zealand Clinical Trials Registry (ANZCTR), with registration no. ACTRN 12621000013864. The Strengthening the Reporting of Observational Studies in Epidemiology (STROBE) standards were followed, and the relevant checklist for enrolment and allocation of participants was used [17]. Written informed consent was obtained from all eligible participants prior to the study.

### 2.2. Ethics

The study protocol was approved by the Bioethics Committee of the Wroclaw Medical University, Poland (approval no. KB–815/2020). The study was carried out according to the Declaration of Helsinki and Good Clinical Practice guidelines. Written informed consent was obtained from all the participants prior to the study.

### 2.3. Participants

The study was carried out on 82 participants, including 47 physicians and 35 nurses. Two study groups were identified. The first group (study group, *n* = 40) included healthcare professionals who had a symptomatic COVID-19 infection with a confirmed real-time reverse transcription-polymerase chain reaction (RT-PCR) test for SARS-CoV-2. The second group (control group, *n* = 42) included medical staff who, until the study, had always obtained a negative result (every 14 days) of the RT-PCR test for SARS-CoV-2. The primary study outcome was to assess serum AT1R levels relative to COVID severity. We also analysed demographic data (age, sex, blood group, and body mass index (BMI)) and collected information on chronic diseases (diabetes, hypertension, nicotinism, and kidney failure) and medications. None of the study participants took AT1R blockers or ACE inhibitors.

### 2.4. Outcomes

#### 2.4.1. AT1R Serum Concentration

On the day of the examination, one sample of blood (2.7 mL) was taken from each patient to determine the AT1R levels. After collection, the blood samples were left at room temperature to clot (about 30 min). They were then centrifuged at 3000 rpm for 10 min. The resulting serum was frozen and stored at −70 °C until the determination was performed. A 96-well plate coated with an anti-human AT1R antibody was used for test purposes. For analysis, 100 µL of the standard solution at 0, 0.156, 0.312, 0.625, 1.25, 2.5, 5, and 10 ng/mL, was added in duplicate. The remaining wells were filled with 100 µL of patient/control sera, added in duplicate. After 2 h of incubation at 37 °C, the liquid from each well was removed. Then, 100 µL of detection reagent A was added to each well and left for 1 h at 37 °C temperature. After 1 h of incubation, the wells were washed three times with 350 µL of a wash buffer. Subsequently, 100 µL of detection reagent B was added to each well and left for 1 h at 37 °C temperature. After the next washing step (five times with 350 µL of the wash buffer), 90 µL of 3,3′,5,5′-tetramethylbenzidine (TMB) was added to each well. After 10 min of incubation at 37 °C temperature, the reaction was stopped with a stop solution. Absorbance was read using a microplate reader Tecan Infinite 200 (Tecan Austria GmbH, Grödig, Austria) at 450 nm. Serum level of AT1R was determined in accordance with the recommendations of the manufacturer Biomatik (Kitchener, Ontario, Canada) no. EKU02409 AT1R ELISA Kit. According to the manufacturer’s description, the detection range is 0.156–10 ng/mL. The minimum detectable dose of AT1R is typically less than 0.055 ng/mL. The sensitivity of this assay, or Lower Limit of Detection (LLD) was defined as the lowest protein concentration that could be differentiated from zero. It was determined by subtracting two standard deviations to the mean optical density value of 20 zero-standard replicates and calculating the corresponding concentration. This assay has high sensitivity and excellent specificity for detection of AT1R. No significant cross-reactivity or interference between AT1R and analogues was observed.

#### 2.4.2. COVID Severity

In the COVID-19-recovered individuals, SARS-CoV-2 infection symptoms and the severity of COVID-19 were additionally evaluated according to the illness categories described in the Clinical Spectrum of SARS-CoV-2 Infection section of the COVID-19 Treatment Guidelines developed by the NIH [3]:

1. Asymptomatic or Presymptomatic Infection: Individuals who tested positive for SARS-CoV-2 using a virologic test (i.e., a nucleic acid amplification test or an antigen test) but had no symptoms that were consistent with COVID-19;
2. Mild Illness: Individuals who had any of the various signs and symptoms of COVID-19 (e.g., fever, cough, sore throat, malaise, headache, muscle pain, nausea, vomiting, diarrhoea, loss of taste and smell) but who did not have shortness of breath, dyspnoea, or abnormal chest imaging;
3. Moderate Illness: Individuals who showed lower respiratory disease evidence during clinical assessment or imaging and had a saturation of oxygen (SpO_2_) ≥ 94% on room air at sea level;
4. Severe Illness: Individuals who had SpO_2_ < 94% on room air at sea level, a ratio of the arterial partial pressure of oxygen to fraction of inspired oxygen (PaO_2_/FiO_2_) < 300 mmHg, a respiratory rate >30 breaths per minute, or lung infiltrates >50%;
5. Critical Illness: Individuals who had respiratory failure, septic shock, and/or multiple organ dysfunction.

### 2.5. Sample Size

Sample size analysis was performed using Statistica 13 (TIBCO Software Inc., Palo Alto, CA, USA). The difference in the serum AT1R level (ng/mL) between the groups of COVID-19-recovered subjects (*n* = 6) and healthy subjects (*n* = 6) was evaluated based on the available preliminary results of the study conducted at our centre (pilot study, *n* = 12). The sample size estimation analysis used the mean scores and standard deviations of the AT1R level (ng/mL) in both groups: the mean score in the COVID-19-recovered group was 3.02 ng/mL (SD = 1.71 ng/mL); the mean score in the group of health subjects was 4.10 ng/mL (SD = 1.79 ng/mL). The estimated sample size was calculated with a two-sample t-test for means (t-test for independent samples). The α level was set at 0.05, and the power of the test was 0.8. We assumed that there was no correlation between the evaluated variables, and a two-sided null hypothesis was adopted. The final sample size was set at *n* = 40 in each group.

### 2.6. Statistical Analysis

The statistical analysis was performed using Statistica 13 (TIBCO Software Inc., Palo Alto, CA, USA). For measurable variables, arithmetic means, medians, quartiles, standard deviations, and the range of variability (extreme values) were calculated. The frequency of the occurrence (percentage) of the qualitative variables was calculated. All the measured quantitative variables were tested with the Shapiro–Wilk test to determine the type of distribution. Qualitative variables were compared between the groups using the chi-square test (χ^2^). The comparison of the results of quantitative variables between the groups was performed using the Student’s t-test for independent samples or the Mann–Whitney U test, depending on the fulfilment of the test assumptions. The receiver operating characteristic (ROC) curve analysis (with Youden’s index) was performed to determine the optimal cut-off level for AT1R to detect the occurrence of COVID-19. Univariable logistic regression was used to evaluate the influence of individual predictor variables in predicting COVID-19 disease. The results were considered statistically significant when the *p*-value was lower than 0.05.

## 3. Results

A group of 82 participants enrolled in the study, including healthy individuals who never had a positive RT-PCR test for COVID-19 (control group, *n* = 42), and individuals who had recovered from COVID-19 (study group, *n* = 40). Women represented 59.75% (*n* = 49) and men 40.25% (*n* = 33). The mean age of the participants was 39.9 years (SD = 9.8 years). Table 1 shows the detailed characteristics of the group together with a comparison of these characteristics between the COVID-19 recovered subjects and the control group. The groups were homogeneous regarding the selected characteristics (Table 1).

### 3.1. Analysis of AT1R Serum Concentration and Selected Values in Both Groups

**Figure 2:**
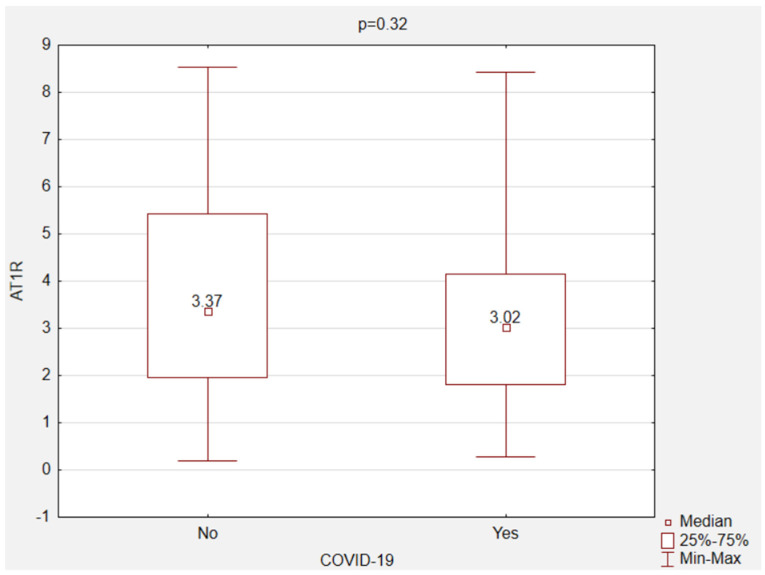
AT1R concentration in the non-COVID-19 and the COVID-19 recovered individuals. Abbreviations: AT1R, angiotensin II type 1 receptor.

**Figure 3:**
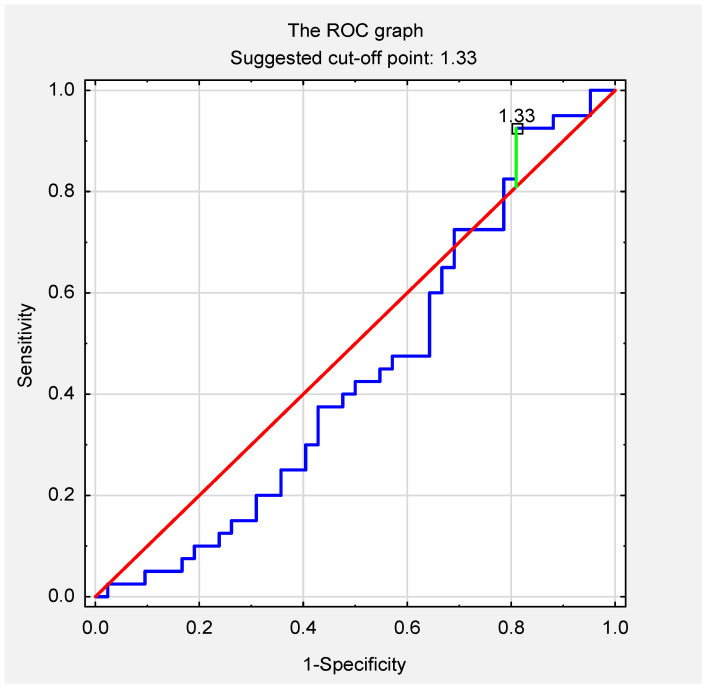
ROC curve for AT1R serum concentration in the non-COVID-19 and the COVID-19 recovered individuals. Abbreviations: ROC, receiver operating characteristic curve.

There were no statistically significant differences in the mean values of AT1R serum concentration in the recovered individuals and the non-COVID-19 subjects (control group) (3.29 vs. 3.76 ng/mL; *p* = 0.32) (Figure 2). The ROC curve for the AT1R assay revealed that the optimal cut-off point was 1.33 (area under the curve, 0.44; 95% confidence interval, 0.32–0.57; *p* = 0.37) (Figure 3). The relationship between the selected variables and AT1R levels was assessed in all subjects. The analysis of the unifactorial logistic regression model did not show a significant statistical relationship between the variables (age, sex, body weight, height, BMI, blood group, Rh factor, hypertension, diabetes, thyroid disease, and COVID-19) and the level of AT1R (Table 2).

### 3.2. Analysis of AT1R Serum Concentration and Selected Variables in the COVID-19 Recovered

An attempt was also made to compare intragroup and intergroup AT1R levels with stratification according to the aforementioned variables. No statistically significant differences were found in the inter- and intra-group comparisons (Table 3). The relationship between the selected variables and the AT1R level in the COVID-19-recovered group was assessed. The univariate logistic regression model showed a statistically significantly lower levels of AT1R in volunteers with blood group A than in those with blood group O. The other variables did not have a statistically significant influence on the level of AT1R (Table 4). Further, in the COVID-19-recovered group, the effect of AT1R serum concentration on the severity of the course was assessed according to the NIH guidelines. There was no statistically significant effect of AT1R serum levels on the severity of SARS-CoV-2 infection (Table 5).

## 4. Discussion

In our study, we found no statistical relationship between AT1R serum levels and the severity of infection based on the NIH COVID-19 Treatment Guidelines [3]. There were no statistically significant differences in the mean AT1R serum levels in the recovered individuals and the non-COVID-19 subjects. There was no statistical relationship between AT1R serum concentration in subjects with comorbidities such as hypertension, type II diabetes, and thyroid disease, or with COVID-19 severity.

The methodology assumes that although the AT1R is a membrane GPCR receptor occurring mainly in the vascular endothelium, part of it may be in the blood, which will allow its detection and quantification. To date, no soluble version of this receptor has been described, however, the available test for the determination of AT1R has been validated for determination in blood serum. Especially in the COVID-19 recovered group due to the significant stimulation of AT1R by AngII, it was assumed that its amount may be higher compared to the non-COVID-19 group. The results did not confirm an increase in receptor concentration in the COVID-19 group. Studies of AT1R concentration were also carried out in other body fluids.

Hu et al. [18] in 2009 determined the concentration of AT1R in the urine of Sugar Rats using immunoblotting method, comparing the results with the number of receptors in renal biopsies. The work does not explain in detail the mechanism of the presence of receptors in the urine but suggest that AT1R can be expressed in vascular tissues throughout the body and may be filtered to some extent through the glomerulus. On the other hand, in the study by Bansal et al. [19], the concentration of the AT1R receptor in serum exosomes was determined by Western blot and densitometry analysis.

Knowing the nature of the AT1R receptor, we are not able describe the mechanism of the appearance of this receptor in the sera we studied. We would point out that the presence of the receptor was determined in both groups: recovered from COVID-19 and patients who did not undergo this disease, and none of the results showed the absence of the receptor in serum.

The RAS system plays a crucial role in SARS-CoV-2 infection. One component of this system, ACE2, functions as a receptor for the virus. In their study, Guzzi et al. [20] indicated that a decrease in ACE2 expression following COVID-19 infection leads to excessive AT1R activation by AngII. Increased AT1R activation exerts proinflammatory, prothrombotic, and pro-apoptotic effects [21]. Inflammation following a cytokine storm is one of the major causes of mortality in SARS-CoV-2 infection. The severity of inflammation is further exacerbated by AT1R activation by AngII [22].

Currently, there are many ongoing studies on the effect of angiotensin receptor blockers (ARBs) on the course of SARS-CoV-2 infection. Several authors have shown that this type of drug has dual-phase effects with possible antagonistic outcomes [23,24]. Dublin et al. [25] suggested that ARBs may be effective in SARS-CoV-2 infection, modifying disease progression. Further, ARBs possess inverse agonist properties that give them an additional pharmacological effect and improve drug efficacy [26]. Given the hypothesis that the severity of inflammation in COVID-19 depends on AT1 receptor stimulation by AngII, drugs that act on AT1R have been proposed as a treatment for COVID-19 [27]. Zhang et al. [28] found that among COVID-19 patients hospitalised with hypertension, patient treatment with ACEI/ARB was related to a lower risk of all-cause mortality.

At present, the use of ARBs in preventing excessive proinflammatory effects of AngII in COVID-19 is quite controversial. The present study did not show a correlation between AT1R levels and the severity of the symptoms associated with COVID-19, which is a consequence of the dynamics of the inflammatory state. Rothlin et al. [29] demonstrated that the use of ARBs in the treatment of COVID-19 should be considered, depending on the stage and severity of disease, rather than as a component of the continuation of antihypertensive treatment in the group of patients with COVID-19.

Notably, our analysis showed a relationship between the blood group and AT1R serum levels in the COVID-19-recovered group. The univariate logistic regression model showed a statistically significantly lower level of AT1R in volunteers with blood group A than in those with blood group O. Studies published so far have shown a protective effect of anti-A antibodies against the intracellular uptake of SARS-CoV-2 [30,31]. Four studies showed a correlation between blood group and severity of COVID-19; five studies did not find any correlation [32,33,34,35,36,37,38,39,40]. Ray et al. [36] published a study on a cohort of 7031 patients with positive tests for SARS-CoV-2. The authors found that individuals with type O blood were less likely to contract SARS-CoV-2 compared to non-type O blood groups. In our study, we found that participants with type A blood had higher levels of AT1R than those with type O blood among patients who had a SARS-CoV-2 infection; however, there was no correlation between AT1R levels and the severity of infection. An updated meta-analysis published in 2021 by Bhattacharjee et al. [41] showed no significant differences in unadjusted mortality or severity outcomes related to COVID-19 illness in patients with blood groups A/AB compared to those with B/O blood groups.

Although the role of the RAS has been extensively studied in COVID-19 patients, there are, unquestionably, information gaps concerning this topic, especially regarding the role of AT1-inverse agonists and their mechanism action in SARS-CoV-2 infection.

### Study Limitations

This study has some potential methodological limitations that need to be mentioned. A foremost limitation is the single-centre nature of the study. In our opinion, thorough multicentre research is needed on the topic. The number of patients enrolled was relatively small, which calls for future studies with a larger population size.

## 5. Conclusions

The serum levels of AT1R did not correlate with the severity of the course of COVID-19 in the healthcare professional sampled in this study. No statistically significant difference in AT1R serum concentration was found between the recovered individuals and the non-COVID-19 subjects. Our univariate logistic regression model showed a statistically significantly lower level of serum AT1R in volunteers with blood group A than in participants with blood group O. Thus, further studies on the influence of the virus on the RAS system and the effect of AT1R-blocking drugs on the disease course are necessary.

## Figures and Tables

**Table 1:** Characteristics of study participants.

	All *n* = 82	COVID-19 Recovered *n* = 40	Non-COVID-19 *n* = 42	*p*
Age (years)				0.58 *
M ± SD	39.9 ± 9.8	39.3 ± 10.7	39.9 ± 9.8	
Me (Q1–Q3)	38.0 (31.0–47.0)	36.0 (29.5–48.0)	38.0 (31.0–47.0)	
Min–Max	25.0–64.0	25.0–64.0	25.0–64.0	
Weight (kg)				0.62 *
M ± SD	77.9 ± 16.8	78.9 ± 18.6	77.0 ± 15.1	
Me (Q1–Q3)	77.5 (67.0–87.0)	79.0 (66.0–89.5)	76.5 (67.0–82.0)	
Min–Max	45.0–135.0	45.0–135.0	50.0–116.0	
Height (cm)				0.43 *
M ± SD	171.5 ± 8.9	172.4 ± 10.0	170.8 ± 7.8	
Me (Q1–Q3)	173.0 (164.0–180.0)	173.0 (163.5–180.5)	172.5 (164.0–176.0)	
Min–Max	155.0–189.0	157.0–189.0	155.0–184.0	
BMI (kg/m^2^)				0.92 *
M ± SD	26.4 ± 4.7	26.4 ± 5.2	26.3 ± 4.3	
Me (Q1–Q3)	26.3 (22.8–29.4)	26.2 (22.8–29.7)	26.3 (23.3–29.0)	
Min–Max	17.4–37.8	17.4–37.8	18.9–36.3	
Sex (female) *n* (%)	49 (60%)	23 (58%)	26 (62%)	0.68 **
Blood group *n* (%)				0.36 **
O	17 (23%)	5 (14%)	12 (31%)	
AB	8 (11%)	5 (14%)	3 (8%)	
A	33 (44%)	17 (49%)	16 (41%)	
B	6 (22%)	8 (23%)	11 (20%)	
Rh factor *n* (%)				0.12 **
Rh −	14 (19%)	4 (11%)	10 (26%)	
Rh +	60 (81%)	31 (89%)	29 (74%)	
Chronic disease *n* (%)				
Hypertension (Yes)	8 (10%)	5 (13%)	3 (7%)	0.41 **
Diabetes (Yes)	2 (2%)	1 (3%)	1 (2%)	0.97 **
Thyroid disease (Yes)	7 (9%)	4 (10%)	4 (7%)	0.64 **

Abbreviations: *n*, number of participants; M, mean; Me, median; Min, minimum value; Max, maximum value; Q1, lower quartile; Q3, upper quartile; SD, standard deviation; *p*, level of statistical significance. Notes: * *t*-test for independent samples; ** χ^2^ test.

**Table 2:** Linear regression analysis between AT1R serum concentration and selected variables in all subjects.

AT1R Level—Linear Regression						
Variables		B	SE	t	*p*-Value	ß
Age		−0.01	0.02	−0.63	0.53	−0.07
Body height		0.01	0.03	0.26	0.79	0.03
Body weight		−0.02	0.01	−1.11	0.27	−0.12
BMI		−0.07	0.05	−1.43	0.16	−0.16
Sex	F	Ref.				
	M	−0.33	0.23	−1.41	0.16	−0.16
Blood group	O	Ref.				
	A	0.22	0.38	0.57	0.57	0.08
	B	−0.18	0.46	−0.39	0.70	−0.06
	AB	0.03	0.60	0.05	0.96	0.01
Rh factor	–	Ref.				
	+	0.18	0.31	0.58	0.57	0.07
COVID-19	No	Ref				
	Yes	−0.23	0.23	−1.01	0.32	−0.11
Hypertension	No	Ref.				
	Yes	−0.16	0.39	−0.42	0.67	−0.05
Diabetes	No	Ref.				
	Yes	0.84	0.75	1.12	0.27	0.12
Thyroid disease	No	Ref.				
	Yes	0.28	0.48	0.58	0.57	0.09

Abbreviations: B, unstandardized regression coefficient B; SE, standard error; t: B/standard error; ß, standardized regression coefficient ß; F, female; M, male.

**Table 3:** Comparison of AT1R serum concentration between the groups depending on the selected variables.

Variable		AT1R Concentration														*p* *
		COVID-19 Recovered (*n* = 40)							Non-COVID-19 (*n* = 42)							
		M	Me	Min	Max	Q1	Q3	SD	M	Me	Min	Max	Q1	Q3	SD	
Sex	M	3.03	2.99	0.28	7.19	1.48	4.32	2.03	3.25	2.74	0.20	6.71	1.52	5.36	2.26	0.63
	F	3.49	3.05	1.35	8.43	2.48	3.95	1.64	4.07	3.44	0.20	8.53	2.86	5.44	2.35	0.30
*p*-value *		0.48							0.37							
Blood group	O	4.02	3.98	1.82	5.55	3.43	5.31	1.52	3.19	3.13	0.49	6.55	1.17	5.45	2.21	1.00
	AB	4.41	3.95	1.33	8.43	2.48	5.88	2.82	2.06	1.96	0.20	4.02	0.20	4.02	1.91	1.00
	A	2.59	2.87	0.88	5.04	1.78	3.05	1.02	4.91	4.90	1.15	8.53	2.68	7.14	2.52	0.08
	B	3.91	3.76	1.35	7.19	2.92	4.69	1.74	2.73	3.35	0.20	3.89	1.95	3.57	1.40	0.96
*p*-value **		0.72							0.42							
Rh	–	3.57	2.88	1.35	7.19	1.56	5.59	2.67	3.13	3.35	0.49	8.13	1.13	3.89	2.30	0.62
	+	3.33	3.05	0.88	8.43	2.34	3.95	1.61	3.91	3.32	0.20	8.53	2.06	5.79	2.39	0.34
*p*-value *		0.98							0.41							
Hypertension	No	3.23	2.99	0.28	8.43	1.80	3.98	1.77	3.86	3.42	0.20	8.53	1.98	5.44	2.28	0.23
	Yes	3.71	3.43	1.78	7.19	1.82	4.33	2.23	2.44	1.13	0.20	5.99	0.20	5.99	3.11	0.37
*p*-value *		0.59							0.31							
Diabetes	No	3.27	2.99	0.28	8.43	1.80	3.98	1.82	3.70	3.32	0.20	8.53	1.96	5.23	2.33	0.41
	Yes	4.33	4.33	4.33	4.33	4.33	4.33	-	5.99	5.99	5.99	5.99	5.99	5.99	-	-
*p*-value *		-							-							
Thyroid disease	No	3.24	2.98	0.28	8.43	1.79	4.15	1.86	3.67	3.42	0.20	8.53	1.91	5.44	2.30	0.42
	Yes	3.79	3.19	2.91	5.88	2.95	4.63	1.41	4.84	3.28	3.11	8.13	3.11	8.13	2.85	0.60
*p*-value *		0.43							0.56							

Abbreviations: *n*, number of participants; M, mean; Me, median; Min, minimum value; Max, maximum value; Q1, lower quartile; Q3, upper quartile; SD, standard deviation; AT1R, angiotensin II type 1 receptor; M, male; F, female; p, level of statistical significance. Notes: * Mann–Whitney U test; ** Kruskal–Wallis test; Bonferroni correction was used for all comparisons.

**Table 4:** Results of the logistic regression for COVID-19 recovered group.

AT1R Level—Linear Regression						
Variables		B	SE	t	*p*-Value	ß
Age		0.00	0.03	0.17	0.86	0.03
Body height		0.01	0.03	0.41	0.69	0.07
Body weight		0.00	0.02	−0.01	0.99	0.00
BMI		−0.01	0.06	−0.22	0.83	−0.04
Sex	F	Ref.				
	M	−0.23	0.29	−0.78	0.44	−0.13
Blood group	0	Ref.				
	A	−1.14	0.41	−2.78	0.009	−0.48
	B	0.18	0.50	0.35	0.73	0.06
	AB	0.68	0.59	1.15	0.26	0.22
Rh factor	−	Ref.				
	+	−0.12	0.46	−0.26	0.79	−0.05
Symptoms	1–2	Ref				
	2–3	−0.15	0.30	−0.49	0.63	−0.08
Hypertension	No	Ref.				
	Yes	0.24	0.44	0.55	0.59	0.09
Diabetes	No	Ref.				
	Yes	0.53	0.92	0.58	0.57	0.09
Thyroid disease	No	Ref.				
	Yes	0.28	0.48	0.58	0.57	0.09

Abbreviations: BMI, body mass index; AT1R, angiotensin II type 1 receptor; M, male; F, female; B, unstandardized regression coefficient; SE, standard error; t: B/standard error; ß, standardized regression coefficient ß.

**Table 5:** Comparison of AT1R serum concentration with illness category according to NIH Treatment Guidelines in COVID-19 recovered individuals.

NIH Illness Category	*n*	AT1R Serum Concentration in COVID-19 Recovered Group (*n* = 40)							*p*
		M	Me	Min	Max	Q1	Q3	SD	
1	1	7.19	7.19	7.19	7.19	7.19	7.19	-	0.21 *
2	25	3.24	2.99	1.35	6.34	2.46	3.95	1.30	
3	10	3.24	3.28	0.28	5.55	1.71	5.04	1.72	
4	4	2.75	1.11	0.35	8.43	0.62	4.88	3.81	
1–2	26	3.40	3.02	1.35	7.19	2.46	3.98	1.49	0.45 **
3–4	14	3.10	3.01	0.28	8.43	1.33	5.04	2.33	

Abbreviations: NIH, National Institutes of Health; *n*, number of participants; M, mean; Me, median; Min, minimum value; Max, maximum value; Q1, lower quartile; Q3, upper quartile; SD, standard deviation; AT1R, angiotensin II type 1 receptor; p, level of statistical significance. Notes: * Kruskal–Wallis test; ** Mann–Whitney U test.

## Data Availability

The data presented in this study are available on request from the corresponding author.
